# Use of biologic agents and methotrexate improves renal manifestation and outcome in patients with rheumatoid arthritis: a retrospective analysis

**DOI:** 10.1007/s10157-021-02160-2

**Published:** 2021-11-30

**Authors:** Masato Sawamura, Naoki Sawa, Masayuki Yamanouchi, Daisuke Ikuma, Akinari Sekine, Hiroki Mizuno, Masahiro Kawada, Rikako Hiramatsu, Noriko Hayami, Eiko Hasegawa, Tatsuya Suwabe, Junichi Hoshino, Kei Kono, Keiichi Kinowaki, Kenichi Ohashi, Yutaka Yamaguchi, Yoshifumi Ubara

**Affiliations:** 1 Nephrology Center and Department of Rheumatology, Toranomon Hospital, Tokyo, Japan; 2 Department of Pathology, Toranomon Hospital, Tokyo, Japan; 3 Okinaka Memorial Institute for Medical Research, Toranomon Hospital, Tokyo, Japan; 4 Department of Human Pathology, Tokyo Medical and Dental University, Tokyo, Japan; 5 Yamaguchi’s Pathology Laboratory, Chiba, Japan; 6 Nephrology Center, Toranomon Hospital, Kajigaya, 1-3-1, Takatsu, Kawasaki, Kanagawa 212-0015 Japan

**Keywords:** Rheumatoid arthritis, Renal manifestation, AA-Amyloidosis, IgA nephropathy, Membranous nephropathy, Nephrosclerosis, Disease-modifying antirheumatic drugs (DMARDs), Biologic agent

## Abstract

**Background and purpose:**

We examined whether advances in treatment strategies from older disease-modifying antirheumatic drugs (DMARDs) to new biologic agents and methotrexate improved renal complications and outcome in patients with rheumatoid arthritis (RA).

**Methods:**

We reviewed records of 156 patients with RA who underwent kidney biopsy at our institute between January 1990 and December 2019. All patients were assigned to one of three periods: period 1, 1990–1999 (*n* = 48); period 2, 2000–2009(*n* = 57); period 3, 2010–2019 (*n* = 51).

**Results:**

Membranous nephropathy, nephrosclerosis, AA-amyloidosis, and IgA nephropathy were the four major renal manifestations of RA. AA-amyloidosis was diagnosed by kidney biopsy in 21 patients: period 1, 7 patients (15%); period 2, 10 patients (18%); and period 3, 4 patients (8%). The 4 patients in period 3 were in the years 2010–2014, and no new case of AA-amyloidosis was recorded from 2015 to 2019. In all 21 of the patients with AA-amyloidosis, neither a biologic agent nor methotrexate was administered. Fifteen of the 21 patients required dialysis, and 13 died in periods 1–3 because of amyloid-related cardiac dysfunction less than 2 years after the initiation of dialysis. Two of them are doing well using biologic agent despite dialysis. The remaining three patients who received a biologic agent or methotrexate does not progress to end-stage renal failure. In addition, the other renal complications showing progression to dialysis also decreased over time.

**Conclusion:**

Advances in treatment strategies have improved renal outcome and reduced mortality in patients with RA.

**Supplementary Information:**

The online version contains supplementary material available at 10.1007/s10157-021-02160-2.

## Introduction

In 1995, mesangial glomerulonephritis, amyloidosis, and membranous nephropathy (MN) were reported to be the main renal manifestations in patients with rheumatoid arthritis (RA) [1]. In 1998, Nakano et al. also reported that mesangial glomerulonephritis, MN, and secondary amyloidosis were the three most common renal diseases [2]. The term mesangial glomerulonephritis was used in the 1990s, but was then replaced by immunoglobulin A (IgA) nephropathy. Disease-modifying antirheumatic drugs (DMARDs), including gold derivatives, D-penicillamine, bucillamine, sulfasalazine, non-steroidal anti-inflammatory drugs (NSAIDs), and steroids were used to treat RA until the 1990s. Thereafter, new antirheumatic drugs were introduced. Methotrexate (MTX) was available in Japan from the late 1990s, and biologic agents, including infliximab, etanercept, and adalimumab, were available from the early 2000s; the biologic agent tocilizumab was introduced in the late 2000s. Advances in treatment strategies resulted in improved outcomes and quality of life for patients with RA. In addition, renal complications improved. However, no large-scale study has investigated changes in renal complications diagnosed by kidney biopsy in patients with RA. Therefore, we retrospectively compared renal complications in patients with RA in three periods in the past 30 years.

## Materials and methods

### Patients

**Fig. 1:**
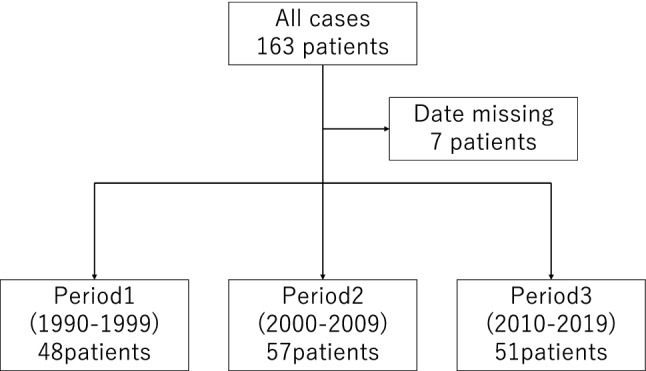
Flow diagram of the study

In this retrospective, single-center cohort study, we reviewed the records of 156 patients with RA who underwent kidney biopsy or autopsy at our nephrology center between January 1990 and December 2019 [3]. Coexisting cases of the other definite autoimmune diseases including systemic lupus erythematosus and systemic sclerosis, and RA were excluded, but although there are a considerable number of mild cases of Sjogren’s syndrome secondary to RA, they could not be excluded and were included in the present study. There were no coexisting cases of polymyositis or dermatomyositis, and RA. We removed seven cases with missing data from the study. All patients were assigned to one of the following three periods: period 1, 1990–1999; period 2, 2000–2009; and period 3, 2010–2019 (Fig. 1, Table 1). As a general rule, the chief physician and nephrologist team decided to perform kidney biopsy on the basis of the following criteria: proteinuria (≥ 0.2 g/day), hematuria (> 5/high power field [HPF]), or renal dysfunction (estimated glomerular filtration rate [eGFR] < 60 ml/min/1.73m^2^).

**Table 1:** Patient characteristics at the time of kidney biopsy

	Total	Period 1 (1990–1999)	Period 2 (2000–2009)	Period 3 (2010–2019)	*p*
Number	156	48	57	51	
Age [years]					
Median (IQR)	64.0 (57.0–70.0)	61.5 (52–66.3)	64.0 (57–70.0)	69.0 (63–74.0)	0.000238
Mean (SD)	63.4 (10.8)	59.0 (9.78)	63.3 (10.8)	67.6 (10.4)	
Sex					
Woman (*n*)	124	41	44	39	0.474
Cre [mg/dL]					
Median (IQR)	0.9 (0.7–1.7)	1.1 (0.8–1.7)	0.8 (0.5–1.7)	1.0 (0.7–1.6)	0.0247
Mean (SD)	1.5 (1.4)	1.8 (1.78)	1.3 (1.24)	1.4 (1.14)	
eGFR [mL/min/1.73m^2^]					
Median (IQR)	55.2 (25.0–81.4)	48.5 (24.4–66.3)	75.0 (26.0–89.0)	53.2 (26.5–69.5)	0.337
Mean (SD)	53.9 (30.4)	47.1 (27.7)	62.1 (33.1)	51.2 (28.0)	
Proteinuria [g/day]					
Median (IQR)	1.0 (0.3–3.2)	0.45 (0.23–2.15)	1.14 (0.48–2.72)	1.47 (0.36–3.82)	0.13
Mean (SD)	2.1 (2.5)	1.92 (2.76)	2.04 (2.25)	2.12 (2.69)	
U-RBC (> 5/HPF)	48	14	16	18	0.691
Duration [year]					
Median (IQR)	7.3 (2.0–20.3)	7 (3.8–17)	6 (1–19)	10 (3–25)	0.462
Mean (SD)	13 (12.8)	11.9 (11.2)	12.2 (13.0)	14.9 (13.9)	
Prednisolone use	108 (69%)	37 (77%)	40 (70%)	31 (61%)	0.212
Biologic agent use	22 (14%)	0	3 (5%)	19 (37%)	4E–08
MTX use	39 (25%)	2 (4%)	14 (25%)	23 (45%)	0.000017
Bucillamine use	52 (33%)	15 (31%)	27 (47%)	10 (20%)	0.00905
d-Penicillamine use	7 (4%)	6 (13%)	1 (2%)	0	0.00522
Gold derivative use	8 (5%)	5 (10%)	3 (5%)	0	0.0645
Sulfasalazine use	19 (12%)	0	8 (14%)	11 (22%)	4E–08

In clinical practice, there are many cases where kidney biopsy is not performed even if they meet the diagnostic criteria. In the case of RA patients, kidney biopsy cannot be performed in patients who cannot assume a prone position suitable for kidney biopsy due to strong body deformity. Therefore, kidney biopsy is limited to patients who can undergo it.

In the 1990s, rheumatology orthopedists were the primary physicians for RA patients in our hospital, but in the 2000s, with the introduction of new rheumatic drugs, rheumatology internists became the primary physicians for RA patients and became more sensitive to renal function and urinary findings.

### Methods

The following clinical data were collected from the medical records at the time of kidney biopsy: disease duration (years); antirheumatic drugs prescribed just before kidney biopsy; blood data, including serum creatinine (g/dL) and eGFR (cre) (ml/min/1.73m^2^), which were measured with a previously reported method [4]; and urine data, such as proteinuria and hematuria. Proteinuria was measured by 24-h urine collection. Hematuria was considered positive when the number of red blood cells per HPF in resuspended urinary sediment was greater than 5/HPF at more than 2 measurements. Antirheumatic drugs included older DMARDs, such as bucillamine, d-penicillamine, gold derivatives (gold sodium thiomalate and auranofin), and sulfasalazine; methotrexate (MTX); tacrolimus; biologic agents; and steroids, such as prednisolone (PSL).

Kidney biopsy samples were processed by light microscopy (LM), immunofluorescence microscopy (IF), and electron microscopy (EM) with standard techniques [5]. Four pathologists assessed the kidney biopsy findings. LM was stained by hematoxylin and eosin (HE), periodic acid-Schiff (PAS), periodic acid-methenamine silver (PAM), Masson’s trichrome, and elastica- or PAM-Masson’s trichrome. IF was performed with staining for IgG, IgA, IgM, C1q, C3, and C4. For the differential diagnosis of renal amyloidosis, enzyme immunoassay of paraffin sections was performed to stain AA, AL (κ/λ), β2MG, and transthyretin. AA-amyloidosis was diagnosed when amorphous deposits in glomeruli and small arteries showed positivity for congo red and apple-green birefringence under polarizing light; immunohistochemical staining was positive for AA but negative for AL (κ/λ), β2MG, and transthyretin; and EM showed randomly arranged fibrils of 7–12 nm in diameter. IgA nephropathy was diagnosed when LM showed mesangial hypercellularity; IF showed granular staining of IgA in the mesangial area; and EM showed electron-dense deposits (EDDs) in the mesangial area. Previous papers reported on the disease entity of mesangial glomerulonephritis, which included IgA nephropathy and non-IgA nephropathy [1, 2]. However, our pathologist team decided to use the disease name IgA nephropathy instead of mesangial glomerulonephritis because we did not detect mesangial glomerulonephritis with IgA-negative electron-dense deposits by EM. Nephrosclerosis was diagnosed in patients with arteriolosclerosis and minor glomerular abnormalities after other diseases were excluded. Another diagnosis was minimal change nephrotic syndrome (MCNS), a glomerular disease characterized by massive proteinuria with no glomerular lesions on LM or no immunoglobulin staining on IF and with podocyte effacement but no electron-dense deposits on EM. The following disease entities were also diagnosed by kidney biopsy: diabetic nephropathy (DN), focal glomerulosclerosis (FGS), membranoproliferative glomerulonephritis (MPGN), ANCA-related crescentic necrotizing glomerulonephritis (ANCA-GN), ANCA-negative crescentic necrotizing glomerulonephritis (ANCA-negative GN), tubulointerstitial nephritis (TIN), IgA vasculitis, anti-glomerular basement membrane disease (GBM), AL-amyloidosis, endocapillary glomerulonephritis (endocapillary GN), thrombotic microangiopathy (TMA), and Alport syndrome.

The study was performed in accordance with the Declaration of Helsinki and its revisions and was approved by the local research ethics board (approval number: 1633). All patients gave written informed consent.

### Statistical analysis

Analyses were performed with the EZR software package (version 3.6.3). Continuous variables were expressed as the median and interquartile range (IQR) according to their distribution. *P* values were calculated with Spearman's rank correlation coefficient for continuous variables, Mann–Whitney *U* test for nominal variables, and Kruskal–Wallis test for comparison between the three groups, all with post hoc Bonferroni correction. *P* < 0.05 was considered statistically significant.

## Results

### Patient characteristics

The median age at the time of kidney biopsy increased across the three periods: period 1, 61.5 years; period 2, 64.0 years old; and period 3, 69.0 years (Table 1). Our analysis of the antirheumatic drugs at the time of kidney biopsy found that the gold-derivative DMARDs, including gold sodium thiomalate and auranofin, were administered mainly in periods 1 and 2, and the DMARD D-penicillamine was administered mainly in period 1. The DMARD bucillamine was developed in Japan and used widely in Japan and Korea in all three periods. MTX use was higher in periods 2 and 3 than in period 1, and biologic agent use was higher in period 3 than in periods 1 and 2. The biologic agents included etanercept and infliximab in period 2, and etanercept, infliximab, tocilizumab, adalimumab, abatacept, golimumab, and certolizumab in period 3. PSL use decreased from 77% of patients in period 1 to 61% of patients in period 3.

### Histological diagnosis by kidney biopsy

**Fig. 2:**
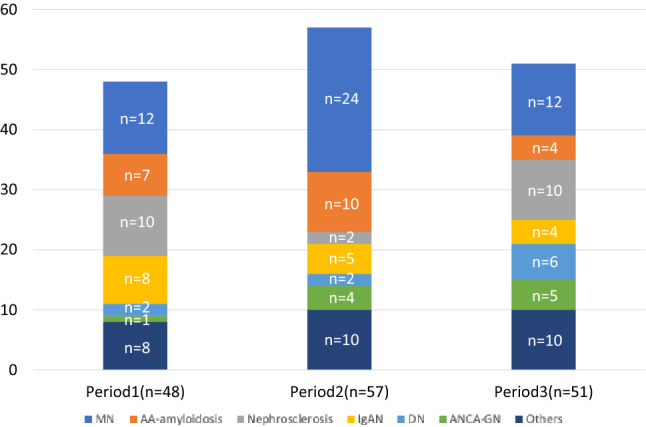
Histological diagnosis by kidney biopsy in the three periods

Histological diagnosis by kidney biopsy is shown in Table 2. The 6 most common renal manifestations were MN (*n* = 48, 30.8%), nephrosclerosis (*n* = 22, 14.1%), AA-amyloidosis (*n* = 21, 13.4%), IgA nephropathy (*n* = 17, 10.9%), ANCA-GN (*n* = 10), and DN (*n* = 10); noteworthy aspects of these 6 diagnoses are described in Table 3. Other diagnoses were MCNS (*n* = 6), FGS (*n* = 5), MPGN (*n* = 4), ANCA-negative GN (*n* = 4), TIN (*n* = 2), IgA vasculitis (*n* = 2), GBM (*n* = 1), AL-amyloidosis (*n* = 1), endocapillary GN (*n* = 1), TMA (*n* = 1), and Alport syndrome (*n* = 1) (Table 2, Fig. 2).

**Table 2:** Histological diagnosis by kidney biopsy and frequency of primary renal disease

		Number	Period 1 (1990–1999)	Period 2 (2000–2009)	Period 3 (2010–2019)
Total	*n*	156	48	57	51
MN	*n* (%)	48 (30.8%)	12 (25%)	24 (43%)	12 (24%)
Primary	*n*	9	1	4	4
Secondary	*n*	39	11	20	8
Gold related	*n*	2	2	0	0
d-penicillamine related	*n*	3	2	1	0
Bucillamine related	*n*	34	7	19	8
Nephrosclerosis	*n* (%)	22 (14.1%)	10(21%)	2 (4%)	10 (20%)
AA-amyloidosis	*n* (%)	21 (13.5%)	7 (15%)	10 (18%)	4 (8%)
IgA nephropathy	*n* (%)	17 (10.9%)	8 (17%)	5 (9%)	4 (8%)
ANCA-GN	*n* (%)	10 (6.4%)	1 (2%)	4 (8%)	5 (10%)
DN	*n* (%)	10 (6.4%)	2 (4%)	2 (4%)	6 (12%)
MCNS	*n* (%)	6 (3.8%)	3 (6%)	2 (4%)	1 (2%)
FGS	*n* (%)	5 (3.2%)	1 (2%)	3 (5%)	1 (2%)
ANCA-negative GN	*n* (%)	4 (2.5%)	1 (2%)	0	3 (6%)
MPGN	*n* (%)	4 (2.5%)	2 (4%)	0	2 (4%)
IgA vasculitis	*n* (%)	2 (1.3%)	0	1 (2%)	1 (2%)
TIN	*n* (%)	2 (1.3%)	1 (2%)	1 (2%)	0
Others		5 (3.2%)	0	GBM (*n* = 1) TMA (*n* = 1) AL-amyloidosis (*n* = 1)	Alport syndrome (*n* = 1) Endocapillary GN (*n* = 1)
Total

**Table 3:** Characteristics of each renal disease

	Number	RA duration (year)	Cre (mg/dL)	eGFR (ml/min/1.73 m^2^)	Proteinuria (g/day)	Hematuria
MN						
Median (IQR)	48	3.5 (1.8–12.8)	0.8 (0.5–0.9)	75.0 (64.0–87.5)	1.9 (0.9–6.2)	6 (13%)
Mean (SD)		8.7 (10.0)	0.8 (0.5)	71.2 (21.3)	3.3 (3.1)	
Nephrosclerosis						
Median (IQR)	22	11.5 (4.3–20.8)	1.0 (0.6–1.4)	53.6 (35.8–71.3)	0.3 (0.1–.4)	3 (14%)
Mean (SD)		14.9 (13.5)	1.1 (0.5)	55.7 (26.0)	0.3 (0.4)	
AA-amyloidosis						
Median (IQR)	21	23.0 (16.0–32.0)	2.7 (1.6–4.2)	14.0 (10.0–23.0)	1.4 (0.3–3.4)	5 (24%)
Mean (SD)		20.4 (11.4)	3.0 (2.0)	26.9 (30.6)	2.0 (1.9)	
IgA nephropathy						
Median (IQR)	17	7.0 (4.0–19.0)	1.1 (0.8–2.0)	48.0 (19.0–57.0)	0.5 (0.3–0.9)	12 (71%)
Mean (SD)		11.6 (12.7)	1.9 (1.9)	42.9 (26.0)	0.8 (1.0)	
ANCA-GN						
Median (IQR)	10	2.0 (1.1–6.5)	1.2 (0.8–1.7)	35.7 (23.6–64.7)	1.3 (0.6–1.9)	9 (90%)
Mean (SD)		8.7 (13.5)	1.5 (1.0)	45.5 (30.1)	1.5 (1.2)	
DN						
Median (IQR)	10	8.0 (3.5–21.8)	1.7 (1.0–2.5)	38.0 (20.1–58.6)	0.5 (0.2–1.2)	1 (10%)
Mean (SD)		13.9 (14.5)	2.1 (1.8)	45.1 (32.1)	1.2 (1.9)	
MCNS						
Median (IQR)	6	4.5 (1.8–5.8)	0.8 (0.6–0.9)	68.5 (62.3–86.0)	6.0 (1.3–7.9)	0
Mean (SD)		3.8 (2.3)	0.7 (0.2)	77.7 (22.9)	4.8 (3.9)	
FGS						
Median (IQR)	5	23.0 (2.0–28.0)	2.0 (1.7–2.8)	22.0 (6.4–24.0)	1.5 (0.8–1.6)	0
Mean (SD)		21.0 (20.7)	2.6 (2.0)	32.5 (29.8)	1.7 (1.3)	
ANCA-negative GN						
Median (IQR)	4	21.0 (8.0–34.3)	1.1 (0.8–1.5)	43.9 (30.5–70.1)	3.5 (1.5–5.5)	3 (75%)
Mean (SD)		21.3 (18.3)	1.1 (0.4)	56.7 (40.4)	56.7 (40.4)	
MPGN						
Median (IQR)	4	10.5 (8.3–13.8)	1.2 (1.0–2.5)	44.0 (26.1–61.8)	2.9 (1.4–4.6)	2 (50%)
Mean (SD)		11.5 (7.9)	2.2 (2.3)	43.9 (30.4)	3.1 (2.1)	
IgA vasculitis						
Median (IQR)	2	27.5 (22.3–32.8)	0.6 (0.6–0.7)	75.6 (72.8–74.8)	1.4 (1.3–1.6)	2 (100%)
Mean (SD)		27.5 (14.8)	0.6 (0.1)	75.6 (7.9)	1.4 (0.5)	
TN						
Median (IQR)	2	8.0 (5.0–11.0)	1.2 (1.2–1.2)	34.6 (33.8–35.3)	0.02 (0.01–0.03)	0
Mean (SD)		8.0 (8.5)	1.2 (0)	34.6 (2.2)	0.02 (0.02)

### Membranous nephropathy (MN)

MN was most common renal disease in every period. Drug-induced MN was found in 39 of the 48 patients with MN (81.3%) (Table 2). It was diagnosed when proteinuria developed after the causal agent was started and subsided after it was discontinued, as described in previous reports [6, 7]. Causal agents included gold derivatives (including gold sodium thiomalate and auranofin), D-penicillamine, and bucillamine. MN related to gold derivatives and D-penicillamine was found mainly in period 1 because use of these drugs decreased after the 2000s (period 2 started in 2000). Bucillamine-related MN was found in all 3 periods because this drug was used consistently throughout them. Idiopathic MN was diagnosed in the remaining 9 patients because proteinuria developed without the administration of one of the causal agents described above. Compared with the other diagnoses, MN had a higher eGFR (median, 75.0 mL/min/1.73m^2^) and shorter RA duration (median 3.5 years).

### Nephrosclerosis

A total of 22 patients were diagnosed with nephrosclerosis. The median duration of RA was 11.5 years, proteinuria was mild (median, 0.3 g/day), and renal dysfunction was also mild (eGFR of median 53.6 mL/min/1.73m^2^) (Tables 2 and 3).

### AA-amyloidosis

A total of 21 patients were diagnosed with AA-amyloidosis by kidney biopsy, as follows: period 1, 7 patients (15%); period 2, 10 patients (19%); period 3, 4 patients (8%). However, in period 3 AA-amyloidosis was found only between 2010 and 2014 and no new cases were diagnosed in the period 2015–2019 (Table 2, Supplemental Fig. 1). In addition, the 21 patients either did not receive MTX or a biologic agent or administration of these drugs was delayed because of an infection. The duration of RA was longer in this disease (median, 21 years) than in the others, and eGFR at the onset was lower (median, 14.0 mL/min/1.73m^2^) (Table 3).

### IgA nephropathy

IgA nephropathy was more common in period 1 than in periods 2 and 3. Hematuria was seen more frequently (71% of patients) than in the other diagnoses (Table 3).

### ANCA-GN

In ANCA-GN, eGFR at the onset was lower than in the other diseases (median 35.7 mL/min/1.73m^2^) (Table 3).

Out of the 14 patients with ANCA-positive (*n*-10) and ANCA-negative (*n* = 4) crescentic necrotizing glomerulonephritis, 1 patient received anti-TNF agents and 3 patients received bucillamine at the time of diagnosis.

### DN

Out of 10 patients with a diagnosis of DN, 5 had a history of long-term steroid use.

### Acute tubulointerstitial nephritis

Acute tubulointerstitial nephritis (ATIN) was diagnosed on 2 patients; In the 1990s, a 61-year-old woman developed AKI after receiving lobenzarit (one of the DMARDs) and improved after discontinuation. Kidney biopsy showed ATIN. In the 2000s, an 80-year-old woman developed AKI after receiving tacrolimus (one of the calcineurin inhibitors) and improved after discontinuation. Kidney biopsy showed also ATIN. Many patients with RA have been treated with concomitant use of non-steroidal anti-inflammatory drugs (NSAIDs), but ATIN induced by NSAIDs was surprisingly not described as a primary diagnosis in our kidney biopsy cohort, although NSAIDs are known to induce ATIN. In addition, chronic tubulointerstitial nephritis associated with long-term use of NSAIDs is known, but it was difficult to determine whether the interstitial damage was caused by NSAIDs alone because many of the patients were elderly and received many other drugs in addition to NSAIDs。

### Relation between renal diseases, dialysis, and outcome

In period 1 (*n* = 48), 14 patients required dialysis: all 7 patients with AA-amyloidosis, 4 out of 8 patients with IgA nephropathy, the 1 patient with FGS, 1 out of 2 patients with DN, and the 1 patient with ANCA-related GN (Table 4).

**Table 4:** Relation between renal diseases and dialysis

	Period 1	Period 2	Period 3
Total	14 (*n* = 48)	11 (*n* = 57)	4 (*n* = 51)
AA-amyloidosis	7 (*n* = 7)	6 (*n* = 10)	2 (*n* = 4)
IgA nephropathy	4 (*n* = 8)	1 (*n* = 5)	1 (*n* = 4)
FGS	1 (*n* = 1)	2 (*n* = 3)	0 (*n* = 1)
DN	1 (*n* = 2)	1 (*n* = 2)	0 (*n* = 6)
ANCA-GN	1 (*n* = 1)	0 (*n* = 4)	1 (*n* = 5)

Each column shows dialysis patent number (total number of each renal disease)

In period 2 (*n* = 57), 11 patients required dialysis: 6 out of 10 patients with AA-amyloidosis, 1 out of 5 patients with IgA nephropathy, 2 out of 3 patients with FGS, and 1 out of 2 patients with DN (Table 4).

In period 3 (*n* = 51), 4 patients required dialysis: 2 out of 4 patients with AA-amyloidosis, 1 out of 4 patients with IgA nephropathy, and 1 out of 5 patients with ANCA-related GN (Table 4).

### Outcome of patients with AA-amyloidosis

In period 1 (*n* = 48), all seven patients with AA-amyloidosis received steroid since after diagnosis, but required dialysis, and died because of amyloid-related cardiac dysfunction less than 2 years after the initiation of dialysis (Fig. 2).

In period 2 (*n* = 58), seven patients with AA-amyloidosis received steroid since after diagnosis. Five patients required dialysis, and died because of amyloid-related cardiac dysfunction less than 2 years after the initiation of dialysis. Two died before initiation of dialysis (one of intestinal perforation, and one suddenly of unknown causes). One patient received a biologic agent since after diagnosis and required dialysis after 9 years, and is doing well more than 2 years even after the initiation of dialysis. The other two, who are receiving MTX and a biologic agent since after diagnosis, are doing well (13 years and 19 years after diagnosis) without any deterioration of renal function (Fig. 2).

In period 3 (*n* = 51), out of two patients who received steroid and did not receive biologic agents because of infectious complication since after diagnosis, one patient died from an infection without receiving a biologic agent, and the outcome of the other is not known. Out of two patients who received biologic agents since after diagnosis, one patient has been doing well for 6 years without any deterioration of renal function. The other received dialysis after 3 years, and is doing well for 2 years by biologic (Fig. 2).

## Discussion

**Fig. 3:**
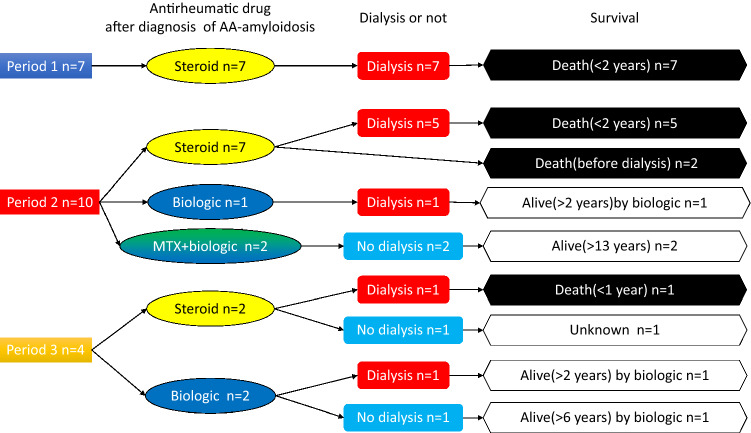
Outcome of patients with AA-amyloidosis

This study retrospectively compared renal complications in patients with RA in the periods 1990–1999, 2000–2009, and 2010–2019. MN, nephrosclerosis, AA-amyloidosis, and IgA nephropathy were the 4 most common renal manifestations of RA over the 30-year period. However, no new case of AA-amyloidosis was recorded from 2015 to 2019. In all diagnoses, progression to dialysis decreased over time (Fig. 3).

Two other representative studies retrospectively examined renal complications of RA. Helin et al. examined kidney biopsy findings in 110 patients with RA during the years 1976–1992 and found that the most common diagnosis was mesangial glomerulonephritis (*n* = 40, 36%) [1]; 8 of these patients were diagnosed with IgA glomerulonephritis, and the remaining 32 patients with non-IgA mesangial glomerulonephritis. The researchers suggested that mesangial glomerulonephritis is associated with long-standing RA and is probably related to the disease itself. Renal amyloidosis was the second most common histopathologic lesion (*n* = 33, 30%). Renal failure was reported as the defining characteristic of renal amyloidosis, but the report did not clarify the presence of the AA subtype [1]. The third most common diagnosis was MN (*n* = 19, 17.2%), which was closely related to DMARDs use in 18 patients (13 patients, gold; 5 patients, d-penicillamine). Nakano et al. also examined kidney biopsy findings in 158 patients in the period 1979–1996 and reported that mesangial glomerulonephritis was the most common renal biopsy finding (*n* = 54, 34.1%) and that 27 of these patients had IgA glomerulonephritis and the remaining 27 had non-IgA mesangial glomerulonephritis [2]. MN was the second most common diagnosis (*n* = 49, 31.0%); it was associated with DMARDs in 40 patients, but the remaining 9 patients did not receive DMARDs. AA-amyloidosis was the third most common diagnosis (*n* = 30, 18.8%) and showed more definite renal dysfunction and a longer duration of RA than the other diagnoses [2].

These two papers [1, 2] suggested the disease entity of mesangial glomerulonephritis was one of the most common renal manifestations of RA. However, current textbooks of renal pathology do not include the disease entity of non-IgA mesangial glomerulonephritis. Our pathologist team did not identify non-IgA mesangial glomerulonephritis in the records of any patients in this study. Therefore, we propose that IgA nephropathy is one of the main renal manifestations of RA and that the diagnosis of mesangial glomerulonephritis should not be used. IgA nephropathy has been reported to have a good renal outcome, but our study found that four out of eight patients with this diagnosis progressed to dialysis. In the majority of patients with RA and MN, the renal complication was reported to be secondary to use of DMARDs, including gold, D-penicillamine and bucillamine [1, 2, 6, 7]. The renal outcome of such drug-induced cases of MN was reported to be good because proteinuria subsided after discontinuation of the causal agent and renal function did not decrease. Nagahama et al. reported that bucillamine-induced MN is characterized by poor spike formation in LM; heterogeneous IgG staining along the glomerular basement membrane in IF; positivity for the IgG subclasses 1, 2, or 3; and small epithelial EDDs in EM [6]. Our study showed that the renal outcome of MN is good, and we did not find any advanced cases leading to renal failure.

AA-amyloidosis has been reported to contribute to renal failure and poor survival [1, 8]. Kuroda et al. reported that patients with amyloidosis have a higher mortality rate but that biologic therapy has a tendency to reduce the risk of dialysis initiation and improve the survival rate in patients with AA-amyloidosis [9]. Our study showed that after introduction of MTX and biologic agents into clinical practice, new cases of AA-amyloidosis decreased and biologic agents prevented the disease progressing to renal failure. Recently, Vinicki et al. reported on kidney biopsy findings from 65 patients with RA in Argentina [10]. They showed a significant reduction in the frequency of kidney biopsy because of changes in the management of RA. Zhang et al. reported on kidney biopsy findings in 56 patients with RA in China from 2007 to 2018 and found that IgA nephropathy was the most common diagnosis (*n* = 27, 48.2%), followed by MN (*n* = 13, 23.2%), and FGS (*n* = 11, 19.6%); AA-amyloidosis was not diagnosed [11]. MTX was used by 13 patients, and a biologic agent was used by 1 patient.

Fewer patients transitioned to dialysis in period 3 than in periods 1 and 2 probably for two reasons: The rate of new onset of AA-amyloidosis decreased, and patients with AA-amyloidosis who were treated with a biologic agent did not progress to end-stage renal failure.

In patients with RA, AA-amyloidosis has been considered to be caused by the accumulation of reactive amyloid protein in the kidneys and other organs as a result of continued chronic inflammation for several decades in a state of poor disease activity. It has been reported that continuous suppression of chronic inflammation not only suppresses the progression of the disease, but also eliminates or reduces it from the tissues [12, 13]. Our study showed that the number of AA-amyloidosis cases decreased after the introduction of newer antirheumatic drugs, and the number of new cases of AA-amyloidosis has also decreased (Tables 2 and 4). This may suggest that suppressing disease activity by curing chronic inflammation for long term not only cures AA-amyloidosis but also prevents it.

IgA nephropathy has been considered to be associated with long-standing RA [1, 2]. However, our study indicates that the frequency of IgA nephropathy seems to be rather low compared to the frequency of detection of IgA nephropathy in general population [14]. The relationship between RA and IgA nephropathy may not be as significant as previously reported.

In conclusion, as treatment strategies for patients with RA have advanced, renal manifestations in RA have changed. New cases of AA-amyloidosis have decreased from 2010 onwards when newer antirheumatic drugs were used, and renal outcome in patients with AA-amyloidosis also improved after 2000. In addition, a decrease was also seen in progression to dialysis with the other renal complications. Although it is difficult to predict the renal prognosis and life outcome of RA patients as a whole because this study was conducted on a very small number of patients who underwent kidney biopsy at our institution, these findings indicate that clinical application of biologic agents and MTX may improve renal outcome and life prognosis in RA patients. This is because with the introduction of newer rheumatic drugs in the 2000s, rheumatologists have shifted from rheumatology orthopedists to rheumatology internists, and have become more sensitive to renal function and urinary findings.

### Limitation

We clarified that newer antirheumatic drugs led to a decrease in new cases of AA-amyloidosis from 2010 onwards by reducing the disease activity, and renal outcome in patients with AA-amyloidosis also improved after 2000.However, we did not clarify the meaning of the other renal manifestation including IgA nephropathy, DMARDs -unrelated MN and nephrosclerosis on patients with RA. In addition, the relation between each disease and management situation of disease activity by using DAS28, CDAI, SDAI or Steinbrocker stage classification was not evaluated enough, because many older cases are included in this study. We believe that these problems will be clarified by a multicenter study.

## Supplementary Information

Below is the link to the electronic supplementary material.

Supplemental Figure 1. Histological diagnosis by kidney biopsy and frequency of primary renal disease (PPTX 43 kb)

## Abbreviations

ANCA-GN: ANCA-related crescentic necrotizing glomerulonephritis
ANCA-negative GN: ANCA-negative crescentic necrotizing glomerulonephritis
DM: Diabetic nephropathy
DMARDs: Disease-modifying antirheumatic drugs
EDDs: Electron-dense deposits
EM: Electron microscopy
eGFR: Estimated glomerular filtration rate
HE: Hematoxylin and eosin
HPF: High-power field
MTX: Methotrexate
IF: Immunofluorescence microscopy
IgA: Immunoglobulin A
LM: Light microscopy
MCNS: Minimal change nephrotic syndrome
MN: Membranous nephropathy
MPGN: Membranoproliferative glomerulonephritis
NSAIDs: Nonsteroidal anti-inflammatory drugs
PAS: Periodic acid-Schiff
PAM: Periodic acid-methenamine silver
PSL: Prednisolone
RA: Rheumatoid arthritis
TIN: Tubulointerstitial nephritis
TMA: Thrombotic microangiopathy
