# Supplementary material for: Use of biologic agents and methotrexate improves renal manifestation and outcome in patients with rheumatoid arthritis: a retrospective analysis
Source: Clin Exp Nephrol. 2021 Nov 30;26(4):341–9. doi: 10.1007/s10157-021-02160-2 (PMC8930889; doi:10.1007/s10157-021-02160-2)
Supplement: Supplementary file 1 — Supplemental Figure 1. Histological diagnosis by kidney biopsy and frequency of primary renal disease (PPTX 43 kb) [file 10157_2021_2160_MOESM1_ESM.pptx]

## Slide 1
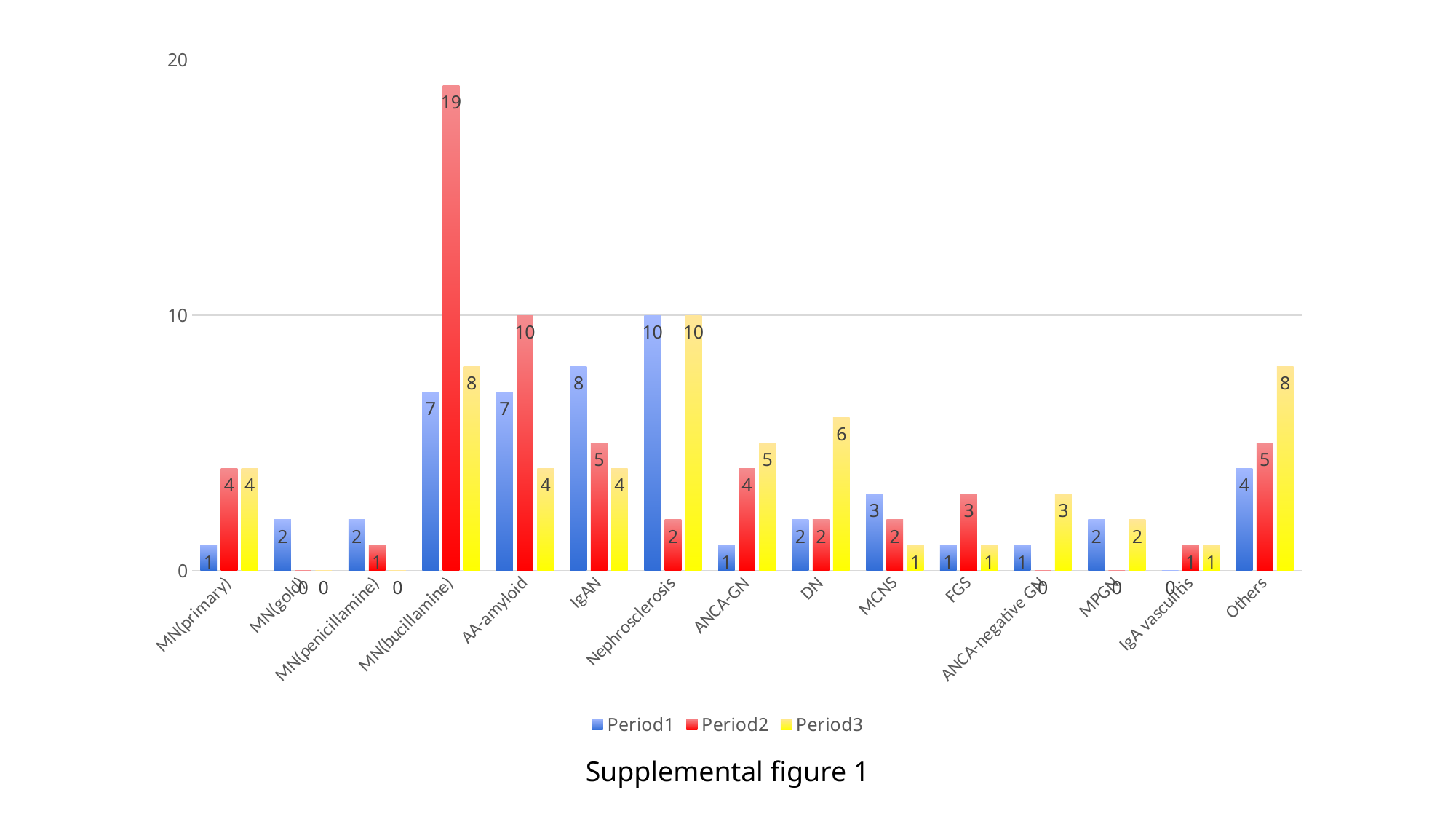

### Chart
| Category | Period1 | Period2 | Period3 |
|---|---|---|---|
| MN(primary) | 1.0 | 4.0 | 4.0 |
| MN(gold) | 2.0 | 0.0 | 0.0 |
| MN(penicillamine) | 2.0 | 1.0 | 0.0 |
| MN(bucillamine) | 7.0 | 19.0 | 8.0 |
| AA-amyloid | 7.0 | 10.0 | 4.0 |
| IgAN | 8.0 | 5.0 | 4.0 |
| Nephrosclerosis | 10.0 | 2.0 | 10.0 |
| ANCA-GN | 1.0 | 4.0 | 5.0 |
| DN | 2.0 | 2.0 | 6.0 |
| MCNS | 3.0 | 2.0 | 1.0 |
| FGS | 1.0 | 3.0 | 1.0 |
| ANCA-negative GN | 1.0 | 0.0 | 3.0 |
| MPGN | 2.0 | 0.0 | 2.0 |
| IgA vasculitis | 0.0 | 1.0 | 1.0 |
| Others | 4.0 | 5.0 | 8.0 |Supplemental figure 1
